# Down-regulation of transforming growth factor-β type II receptor (TGF-βRII) protein and mRNA expression in cervical cancer

**DOI:** 10.1186/1476-4598-7-3

**Published:** 2008-01-09

**Authors:** Jose Diaz-Chavez, Rogelio Hernandez-Pando, Paul F Lambert, Patricio Gariglio

**Affiliations:** 1 Departamento de Genética y Biología Molecular, Centro de Investigación y de Estudios Avanzados, México D.F. 07000, México; 2 Departamento de Patología, Instituto Nacional de Ciencias Médicas y Nutrición Salvador Zubirán, México, D.F. 14000, México; 3 McArdle Laboratory for Cancer Research, University of Wisconsin School of Medicine and Public Health, Madison, Wisconsin 53706, USA

## Abstract

**Background:**

Cervical carcinogenesis is a multistep process initiated by "high risk" human papillomaviruses (HR-HPV), most commonly HPV16. The infection *per se *is, however, not sufficient to induce malignant conversion. Transforming Growth Factor β (TGF-β) inhibits epithelial proliferation and altered expression of TGF-β or its receptors may be important in carcinogenesis. One cofactor candidate to initiate neoplasia in cervical cancer is the prolonged exposure to sex hormones. Interestingly, previous studies demonstrated that estrogens suppress TGF-β induced gene expression. To examine the expression of TGF-β2, TGF-βRII, p15 and c-myc we used *in situ *RT-PCR, real-time PCR and immunohistochemistry in transgenic mice expressing the oncogene E7 of HPV16 under control of the human Keratin-14 promoter (K14-E7 transgenic mice) and nontransgenic control mice treated for 6 months with slow release pellets of 17β-estradiol.

**Results:**

Estrogen-induced carcinogenesis was accompanied by an increase in the incidence and distribution of proliferating cells solely within the cervical and vaginal squamous epithelium of K14-E7 mice. TGF-β2 mRNA and protein levels increased in K14-E7 transgenic mice as compared with nontransgenic mice and further increased after hormone-treatment in both nontransgenic and transgenic mice. In contrast, TGF-βRII mRNA and protein levels were decreased in K14-E7 transgenic mice compared to nontransgenic mice and these levels were further decreased after hormone treatment in transgenic mice. We also observed that c-myc mRNA levels were high in K14-E7 mice irrespective of estrogen treatment and were increased in estrogen-treated nontransgenic mice. Finally we found that p15 mRNA levels were not increased in K14-E7 mice.

**Conclusion:**

These results suggest that the synergy between estrogen and E7 in inducing cervical cancer may in part reflect the ability of both factors to modulate TGF-β signal transduction.

## Background

Cervical cancer (CC) is one of the most frequent cancers affecting women worldwide and is an important public health problem for adult women in developing countries [1].

Infection with HR-HPV types, in particular HPV16 and HPV18, is a crucial step in the etiology of CC [2,3]. The oncogenic process is mainly driven by the viral proteins E6 and E7, which inactivate tumor suppressor gene products p53 and pRB, respectively. Despite infection with HR-HPV subtypes, most precancerous cervical lesions termed cervical intraepithelial neoplasia (CIN) do not progress to in situ or invasive carcinoma implicating either environmental or genetic cofactors in those rare cases where progression occurs [4]. For example, both cigarette smoking and genetic predisposition have been linked to cervical carcinogenesis associated with HR-HPV [5]. Another cofactor that has been repeatedly associated with cervical neoplasia is exposure to estrogen [6]. This raises the important question of which genetic or biological characteristics distinguish CIN lesions that will progress to cancer from the majority that spontaneously regress.

A particularly interesting biological characteristic associated with malignant progression of cervical epithelial cells is their progressive loss of responsiveness to TGF-β [7,8]. TGF-β belongs to a multifunctional family of growth factors that tightly regulate basic cellular functions such as proliferation, apoptosis, differentiation, extracellular matrix turnover and immunosuppression [9].

There are three isoforms of TGF-β: TGF-β1, TGF-β2, and TGF-β3. Each isoform is encoded by a distinct gene, but aminoacid sequences of the three isoforms are 70–80% homologous [10]. TGF-β1 is expressed in endothelial, hematopoietic, and connective tissue cells, TGF-β2 in epithelial and neuronal cells, TGF-β3 primarily in mesenchymal cells [11]. TGF-β2 is an important regulator of differentiation [12] and this function is blocked by E6 and E7 oncoproteins [13,14]. Primary cervical keratinocytes that are immortalized by HPV in vitro and are passaged in culture for prolonged periods of time, eventually lose their sensitivity to the inhibitory effects of TGF-β [15]. In addition, some cell lines derived from CIN lesions are sensitive to TGF-β, whereas lines derived from invasive CCs are resistant [7,8].

The biological effects of TGF-β are primarily mediated by a complex of two transmembrane serine/threonine kinases, the type I (TGF-βRI) and type II (TGF-βRII) receptors [9]. TGF-β signaling cascade is activated when TGF-β binds to TGF-βRII, then receptor I is recruited into the complex and phosphorylated by receptor II at serine and threonine residues [16]. Activated TGF-βRI phosphorylates Smad2 and/or Smad3, and a heterotrimeric complex is formed with Smad4 that translocates into the nucleus, binds a consensus sequence, and directly or indirectly (by interacting with other transcription factors) regulates gene transcription [9].

TGF-β induces growth inhibition of most cell types by causing arrest in the G1 phase of the cell cycle. In normal epithelial cells, TGF-β has been shown to induce the expression of the cyclin-dependent kinase (CDk) 4/6 inhibitor p15Ink4B (p15) [17] and repress the expression of c-Myc [18]. In certain cell types, TGF-β also upregulates p21 [19], a CDK2 inhibitor and downregulates cdc25A, a phosphatase that activates CDK2 [20]. Induction of CDK inhibitors appears to represent key events in TGF-β induced growth arrest.

Kang et al. [7] examined the expression and structural integrity of TGF-βRI and TGF-βRII genes in a serie of 8 human CC cell lines. Two of these lines failed to express TGF-βRII-specific RNA, which in one case was due to a homozygous gene deletion. In addition, missense mutations, gross rearrangements, truncated or decreased transcripts and aberrant 5'-CpG methylation in the TGF-βRII promoter have been found for this gene in a variety of tumor types [21,22]. Because of the strongly suggestive evidence that CC is associated with loss of TGF-β responsiveness and because cervical epithelial differentiation is altered by E7 in the absence or presence of exogenous estrogen [6,23], we investigated the status of TGF-β2 and TGF-βRII expression in transgenic mice expressing the oncogene E7 of HPV16 under control of the human Keratin-14 promoter (K14-E7 transgenic mice) and nontransgenic control mice treated with slow release pellets of 17β-estradiol. In this animal model E7 and estrogen synergize to induce CC [24]. In comparison with cervical tissue from estrogen-treated nontransgenic mice, we found higher expression of TGF-β2 mRNA and protein in cervical tumors from K14-E7 mice treated with estrogen. In contrast, a significant decrease in TGF-βRII mRNA and protein was detected in the CC arised in estrogen-treated K14-E7 transgenic mice. These results suggest that cervical cancer in estrogen-treated K14-E7 mice is accompanied by elevation of TGF-β2 and reduction of TGF-βRII expression.

## Results

### Histological features of cervical epithelium from untreated and estrogen-treated K14-E7 transgenic mice compared with nontransgenic mice

We evaluated the histopathology and expression of TGF-β2 and TGF-βRII at the transcription and protein levels by in situ RT-PCR, real-time PCR and immunohistochemistry respectively in a total of 40 mouse cervical tissues. Ten samples each from nontransgenic (Nt-E), 17β-estradiol treated (6 months) nontransgenic (Nt+E), untreated K14-E7 transgenic (E7-E) and estrogen-treated K14-E7 transgenic (E7+E) mice were employed in this study.

**Figure 1:**
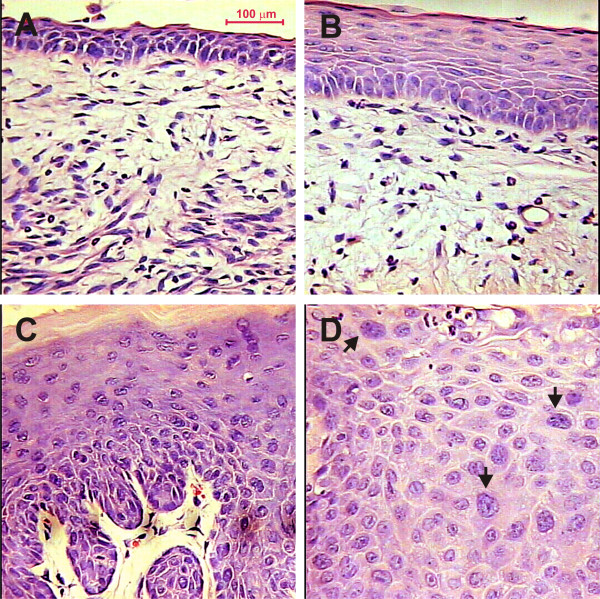
**Representative histological features of cervical epithelium from untreated and estrogen-treated K14-E7 transgenic mice compared with nontransgenic mice**. We evaluated the epithelial histopathology in a total of 40 mouse cervical tissues. Ten samples each from untreated nontransgenic (Nt-E), 17β-estradiol treated (6 months) nontransgenic (Nt+E), untreated K14-E7 transgenic (E7-E) and estrogen-treated K14-E7 transgenic (E7+E) mice were included in this study. Control Nt-E mice show thin cervical epithelium (A). In contrast, Nt+E mice exhibit wider cervical epithelium (B). Thick cervical epithelium constituted by cells with slight dysplasic changes are apparent in E7-E mice (C). In comparison, cervical epithelium from E7+E mice show lack of differentiation with evident dysplastic changes (D) (indicated by black arrows). (All micrographs at 400× magnification, H-E stained cross sections).

The histopathology study revealed that none of the untreated mice developed epithelial cancers of the reproductive tract (Figure 1A). However the squamous epithelium from vagina and cervix in E7-E mice (Figure 1C) was different from their nontransgenic counterparts, due to epithelial rete ridges elongation and rounded basal-like cells with high nuclear/cytoplasmic ratio within the suprabasal compartment, suggestive of differentiation delay. These observations are consistent with ones made previously in this animal model [6], in which, basal-like cells within the suprabasal compartment of untreated K14-E7 cervix were able to perform DNA synthesis, which further strengthen the conclusion that E7 delays differentiation of cervical epithelial cells. Furthermore, double and multinucleated cells were more prevalent in the K14-E7 mice compared with nontransgenic mice, consistent with the ability of E7 to induce endoreduplication [25]. As previously described [24], E7+E mice displayed evidence of high-grade dysplasia and CC (Figure 1D). In contrast, the squamous epithelium from Nt+E mice only showed epithelial hyperplasia without evidence of dysplasia (Figure 1B). It is important to mention that invasive cancer or high grade cervical lesions (CIN III or CIS) occurred in 100% of E7+E mice [24].

### Detection and quantification of TGF-β2 and TGF-βRII mRNA by *in situ *RT-PCR and real-time PCR in cervix of nontransgenic and K14-E7 transgenic mice

**Figure 2:**
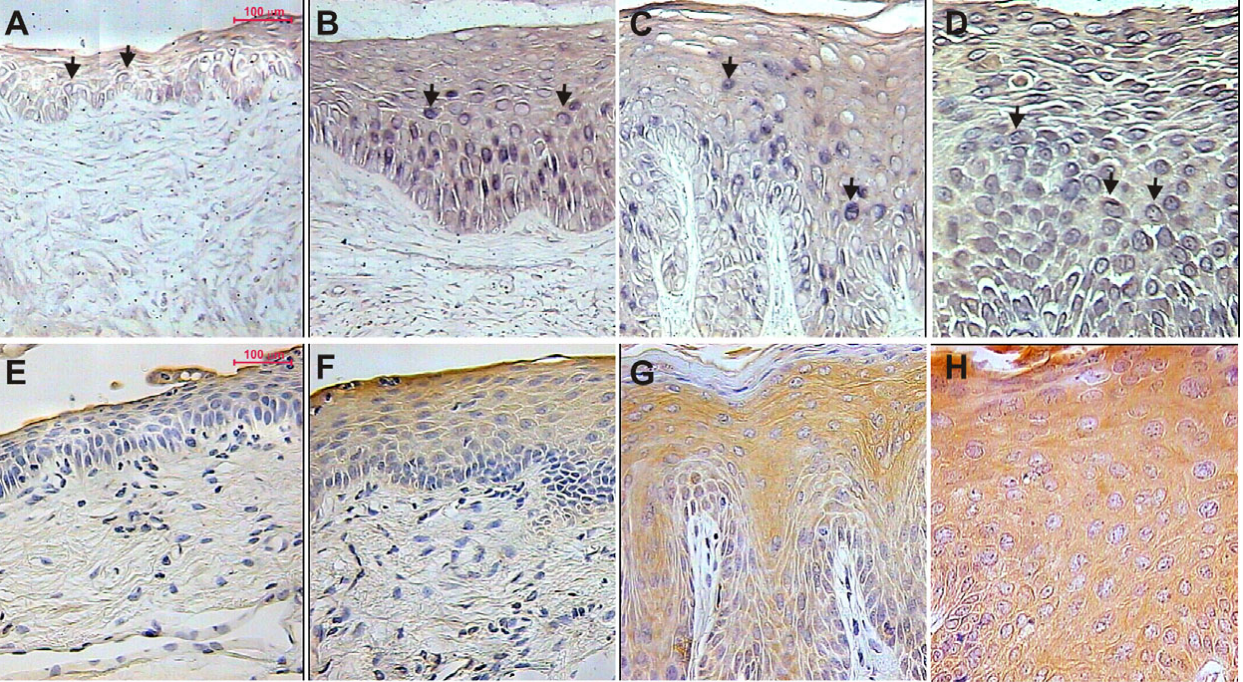
**Representative in situ RT-PCR (A-D) and immunohistochemical (E-H) detection of TGF-β2 in cervical tissue**. Shown are histological cross-sections of the mouse cervical tract from Nt-E, Nt+E, E7-E and E7+E mice. Figure 2A and E: Nt-E mice; (B and F): Nt+E mice; (C and G): E7-E mice; (D and H): E7+E mice. In both cases (mRNA, protein), signal was mainly cytoplasmic (indicated by black arrows for *in situ *RT-PCR). TGF-β2 mRNA and protein levels were increased throughout multiple layers of cells in LSILs (C and G) and CIS (D and H) arising from K14-E7 transgenic mice, whereas weaker TGF-β2 mRNA and protein expression was detected in the normal cervical squamous epithelium (A and E) and hyperplastic lesions (B and F) arising from nontransgenic mice. These experiments are representative of five separate experiments (all micrographs at 400× magnification).

*In situ *RT-PCR analysis (Figure 2A–D) demonstrated an increase of TGF-β2 mRNA in the cervical squamous epithelium from estrogen-treated mice (Figure 2B and 2D) compared to untreated controls (Figure 2A and 2C). Additionally, we observed that the level of TGF-β2 mRNA in E7-E mice was higher than in Nt-E mice (Figure 2A and 2C). E7+E mice displayed the highest in situ RT-PCR signals of all four sample sets (Figure 2D). Together these results indicate that the TGF-β2 mRNA is upregulated in its expression by both estrogen and E7.

**Figure 3:**
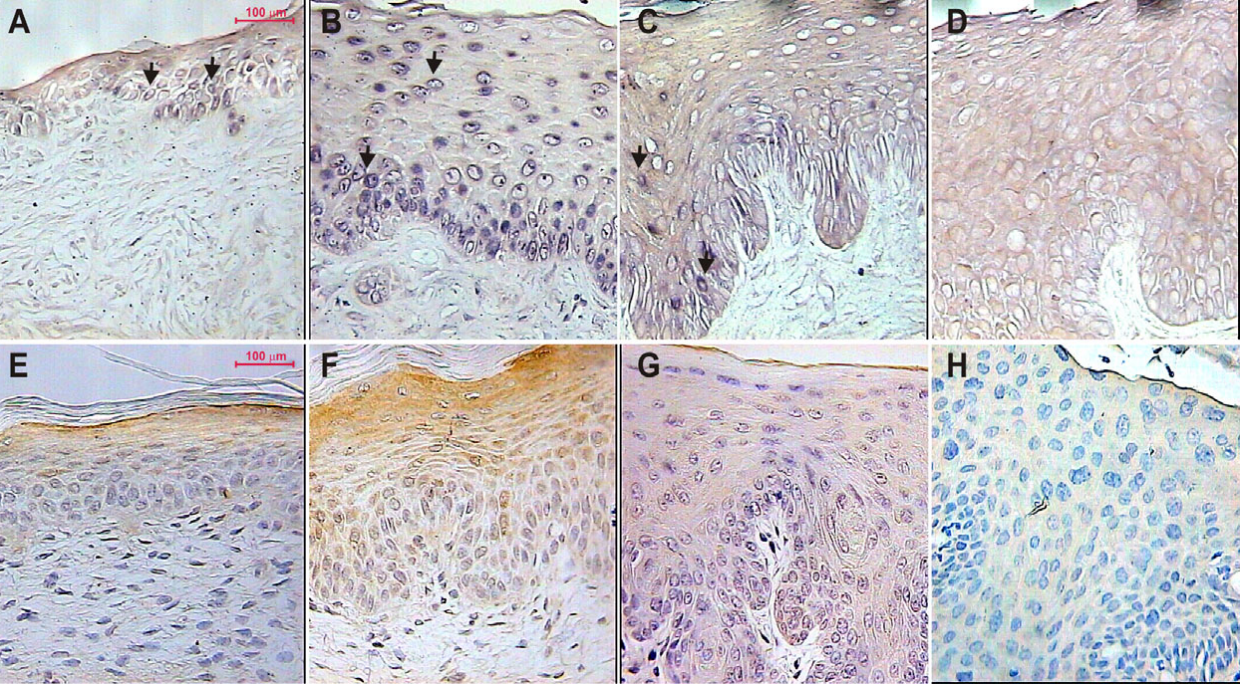
**Representative *in situ *RT-PCR (A-D) and immunohistochemical (E-H) determination of TGF-βRII in cervical tissue**. Shown are histological cross-sections of the mouse cervical tract from Nt-E, Nt+E, E7-E and E7+E mice. Figure 3A and E: Nt-E mice; (B and F): Nt+E mice; (C and G): E7-E mice; (D and H): E7+E mice. In both cases (mRNA, protein), signal was mainly cytoplasmic (indicated by black arrows for *in situ *RT-PCR). TGF-βRII mRNA and protein expression was increased in hyperplastic lesions arising from Nt+E mice (B and F) but note that staining was lower in E7-E (C and G) and E7+E mice (D and H) in comparison with Nt-E mice (A and E). These experiments are representative of five separate experiments (all micrographs at 400× magnification).

We next analysed expression of the TGF-β type II receptor (Figure 3). Compared with untreated control, TGF-βRII mRNA expression was increased in estrogen-treated nontransgenic mice (Figure 3A and 3B), but interestingly the expression of this receptor was decreased in E7+E mice in comparison to its untreated K14-E7 control mice (Figure 3C and 3D). Thus signal in E7+E mice was the lowest among analysed cervical samples (Figure 3D).

As a control for the DNase treatment, DNA signal was detected only in cell nuclei on control slides that were not pretreated with DNase (data not shown). No signal was detected in the cytoplasm of control slides, in which RT-PCR was performed without reverse transcriptase or with only one oligonucleotide (data not shown).

**Figure 4:**
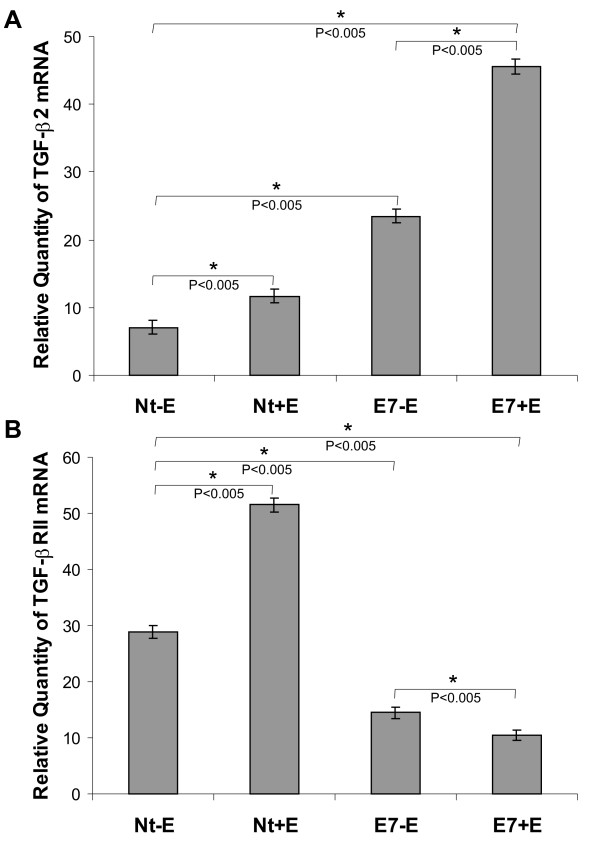
**Quantification of TGF-β2 and TGF-βRII gene expression using real-time PCR in cervical tissue including the transformation zone**. We evaluated TGF-β2 and TGF-βRII mRNA in a total of 3 mice from each group. Samples from the following mice categories were employed: Nt-E, Nt+E, E7-E and E7+E mice. (A) Quantification of TGF-β2 mRNA. The data are presented as the fold change in TGF-β2 mRNA level normalized to the gapdh mRNA (endogenous control). Nt-E mice were used as calibrator. TGF-β2 mRNA levels were increased in estrogen-treated mice (Nt+E and E7+E) in comparison with controls (Nt-E and E7-E) (*P *< 0.005). The amount of TGF-β2 mRNA is also increased in E7-E transgenic mice compared with Nt-E (*P *< 0.005) with the highest levels found in E7+E mice. The difference in expression of TGF-β2 mRNA between Nt-E vs Nt+E, E7-E and E7+E mice was statistically significant (*P *< 0.005). Particularly, the difference between Nt-E vs Nt+E was statistically significant (*P *< 0.005). All significant differences are indicated by asterisks. (B) Quantification of TGF-βRII mRNA. The data are presented as the fold change in TGF-βRII mRNA level normalized to the gapdh mRNA (endogenous control). Nt-E mice were used as calibrator. TGF-βRII mRNA expression was increased in Nt+E mice in comparison with Nt-E, E7-E and E7+E transgenic mice. The lowest TGF-βRII mRNA expression level was observed in E7+E mice (*P *< 0.005). The difference in expression of TGF-βRII mRNA between Nt-E vs Nt+E, E7-E and E7+E mice was statistically significant (*P *< 0.005). Specifically, the difference between E7-E vs E7+E was statistically significant. All significant differences are indicated by asterisks.

The expression levels of TGF-β2 and TGF-βRII mRNA determined by *in situ *RT-PCR were confirmed by real-time PCR in cervical tissue including the transformation zone. Using the 2^ΔΔCT ^method, the change in TGF-β2 and TGF-βRII gene expression of cervical samples was normalized to gapdh mRNA. In comparison with Nt-E mice, TGF-β2 mRNA levels were increased 1.5 fold and 5.6 fold in Nt+E and E7+E mice respectively (*P *< 0.005) (Figure 4A). The amount of TGF-β2 mRNA was also increased 3 fold in E7-E compared with Nt-E mice (*P *< 0.005) (Figure 4A). TGF-βRII mRNA expression was increased 1.7 fold in Nt+E in comparison with Nt-E mice (*P *< 0.005). Cervical samples from K14-E7 transgenic mice show half TGF-βRII mRNA level than nontransgenic mice in the absence of hormone supply and 3 fold less than the level observed in E7+E mice (*P *< 0.005) (Figure 4B). These results suggest that high TGF-β2 mRNA levels and low TGF-βRII mRNA levels are associated with in situ carcinoma arising from estrogen-treated K14-E7 transgenic mice.

### Quantification of c-myc and p15 mRNA in cervix of nontransgenic and K14-E7 transgenic mice

**Figure 5:**
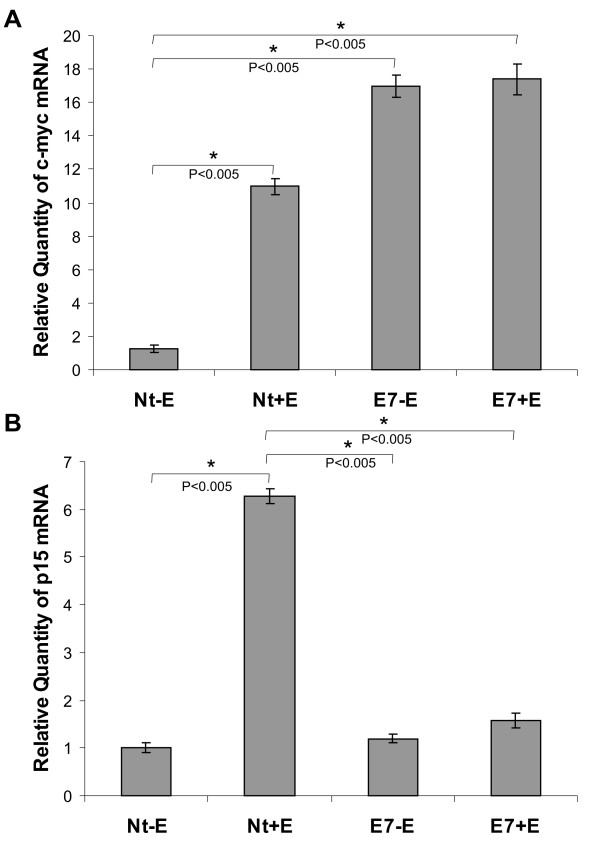
**Quantification of TGF-β target genes c-myc and p15 gene expression using real-time PCR in cervical tissue**. We evaluated c-myc and p15 mRNA in a total of 3 mice from each group. Samples from the following mice categories were employed: Nt-E, Nt+E, E7-E and E7+E mice. (A) Quantification of c-myc mRNA. The data are presented as the fold change in c-myc mRNA level normalized to the gapdh mRNA (endogenous control). Nt-E mice were used as calibrator. c-myc mRNA levels were very high in K14-E7 mice irrespective of estrogen treatment and were strongly increased in Nt+E mice as compared with the control Nt-E (*P *< 0.005) (Figure 5A). The difference in expression of c-myc mRNA between Nt-E vs Nt+E, E7-E and E7+E mice was statistically significant (*P *< 0.005). All significant differences are indicated by asterisks. (B) Quantification of p15 mRNA. The data are presented as the fold change in p15 mRNA level normalized to the gapdh mRNA (endogenous control). p15 mRNA levels were not increased in K14-E7 mice even in the presence of the hormone; however p15 mRNA levels were very high in Nt+E mice (*P *< 0.005) (Figure 5B). The difference in expression of p15 mRNA between Nt-E vs Nt+E, and Nt+E vs E7+E mice was statistically significant (*P *< 0.005). There was not statistical significance between E7-E vs E7+E P > 0.005. All significant differences are indicated by asterisks.

In normal conditions, the TGF-β pathway leads to downregulation of c-myc expression and upregulation of cdks inhibitors, such as p15 and p21 [26]. The repression of c-myc has been shown to be required for the induction of p15 by TGF-β [27] and it was previously reported that loss of c-myc repression is central to TGF-β resistance mechanism [26]. We therefore examined whether or not c-myc and p15 expression was different in cervical samples from K14-E7 transgenic and nontransgenic mice and the effect of 17β-estradiol treatment. We found that c-myc mRNA levels were higher in K14-E7 mice irrespective of estrogen treatment and were increased in estrogen-treated nontransgenic mice (Figure 5A). p15 mRNA levels were not increased in K14-E7 mice even in the presence of the hormone; however p15 mRNA levels were very high in Nt+E mice (Figure 5B).

### Detection of TGF-β2 and TGF-βRII protein in cervix of nontransgenic and K14-E7 transgenic mice by immunohistochemistry

As *in situ *RT-PCR and real-time PCR showed upregulation of TGF-β2 mRNA in estrogen-treated nontransgenic and K14-E7 mice and downregulation of TGF-βRII mRNA in both E7-E and E7+E mice, we performed immunohistochemistry to assess if there exists a correlation between mRNA and protein expression of these genes.

The immunoreactivity against TGF-β2 was increased in Nt+E and K14-E7 transgenic mice in comparison with respective untreated controls (Figure 2). It was also evident that TGF-β2 protein levels were higher in cervix of K14-E7 transgenic mice (Figure 2G and 2H) compared with nontransgenic controls (Figure 2E and 2F), particularly in E7+E mice (Figure 2G).

TGF-βRII immunostaining was higher in Nt+E mice in comparison with untreated nontransgenic animals (Figure 3E and 3F). In contrast, TGF-βRII staining was lower in E7+E mice (Figure 3H) when compared to E7-E mice (Figure 3G). The lowest level of this important receptor was detected in E7+E mice (Figure 3H). Therefore, it seems that there is a correlation between higher expression of TGF-β2 and decrease of TGF-βRII expression in this particular model of cervical carcinogenesis, suggesting TGF-β2 participation in the loss of growth control and promotion of tumorigenesis.

## Discussion

In several cancer types, there is a strong correlation between malignant progression and loss of sensitivity to the antiproliferative effects of TGF-β, which is frequently associated with reduced expression or mutational inactivation of TGF-β receptors [28,29]. Interestingly, re-expression of TGF-βRII restored response to exogenous TGF-β and reversed the malignant behavior of various cell lines [30]. We demonstrate here a diminished expression of TGF-β type II receptor in CC at the mRNA and protein levels. Such decrease in TGF-βRII expression might lead to loss of TGF-β sensitivity in CC cells from our HPV-transgenic mouse model, and ultimately to escape from growth regulatory signals imposed by TGF-β.

TGF-β2 shares 71% homology in the amino acid sequence with TGF-β1, and they are interchangeable in bioassays [31]. Overproduction of TGF-β has been associated with tumors of many histologic types including those of breast, prostate, lung, liver and colon [11]. These high TGF-β levels in tumor tissues, including cervical cancer, correlate with markers of higher metastatic phenotype and/or poor patient outcome [32], and many tumor cells exhibit increased invasiveness in response to TGF-β [33]. In addition, TGF-β2 was higher expressed than TGF-β1 and TGF-β3 in tumor cells of malignant HaCaT-Ras clones, particularly at the invasion front [34]. TGF-β can also induce an epithelial-to-mesenchymal transition in tumor and nontumor epithelial cells [35], and it has been demonstrated that TGF-β1 stimulates epithelial-mesenchymal transition in SiHa (HPV16) cells [36]. Other recent studies demonstrated that TGF-β is overexpressed in Pap smears correlating with CIN progression to cancer [37,38]. Even more, it was found that specific TGF-β2 expression is a common feature in CIN lesions [39], and it was positively correlated with E7 expression [22]. Moreover, HPV16 E6 and E7 increased TGF-β1 promoter activity [40]. Our results confirm and extend these observations, showing high TGF-β2 mRNA and protein expression in cervical tumors from estrogen-treated K14-E7 mice. A mechanism by which TGF-β2 overproduction may contribute to cervical carcinogenesis is through inhibition of the immune response [41]. However, there are also some studies that disagree with these observations. Decrease of TGF-β1, TGF-β2 and TGF-β3 mRNA expression was reported in some patients with CIN, but that result was not statistically significant [42]. Another study found specific decrease of TGF-β1 expression in CIN I-III, but they did not analyze TGF-β2 [43,44]. Nees et al. [12], found that E7 downregulate the expression of TGF-β2; a possible reason for this apparent contradiction may be that they used primary human keratinocytes while we used a cervical cancer mouse model.

One characteristic associated with malignant progression of cervical epithelial cells is their progressive loss of responsiveness to TGF-β [7,8]. Furthermore, resistance to TGF-β that is acquired by several cell lines correlates with HPV tumorigenic potential. Such studies indicate that after HPV infection, additional cellular or molecular changes might participate in the loss of TGF-β responsiveness, which then promotes malignant transformation. Our results agree with these observations, and suggest that one significant mechanism is the lack of TGFβRII expression.

The antimitogenic action of TGF-β in epithelial cells involves the expression of CDK inhibitors like p15 and down-regulation of c-Myc expression. The significance of c-Myc downregulation in TGF-β action is underscored by the observation that overexpression of exogenous c-Myc renders cells resistant to the antimitogenic TGF-β effect [27]. Particularly, Kim et al., [8] observed in cervical cell lines that growth inhibition by TGF-β includes downregulation of c-myc gene expression. We observed that c-myc mRNA levels were higher in K14-E7 mice irrespective of estrogen treatment, and were also increased in estrogen-treated nontransgenic mice, while p15 mRNA levels were not significatively increased in K14-E7 mice, even in the presence of estrogens. However, p15 mRNA levels were highest in estrogen-treated nontransgenic mice. These results suggest that in our CC model, c-myc levels are increased due to E7 presence and they are not regulated by estrogen.

Estrogen contributes not only to the onset, but also to the persistence and malignant progression of CC in our HPV-transgenic mouse model [6], and this is supported by epidemiological evidence in humans [45]. One notable finding in our study is that in estrogen-treated K14-E7 mice it was observed the lowest TGF-βRII levels as compared to nontransgenic mice. Our results indicate that this striking down-regulation of TGF-βRII expression is at the transcriptional level. In spite of the enormous amount of work that has been published on the role of estrogen in hormonal carcinogenesis [46], the mechanism by which estrogen contributes to cervical cancer is not clear, but could be an initiation factor of neoplastic lesions acting as a direct carcinogen [46].

Chronic elevation of estrogen levels by oral contraceptive use or during pregnancy, might be sufficient to contribute to tumor growth in HR-HPV infected women. The high frequency of spontaneous regression of high-grade cervical dysplasia in women, is consistent with suboptimal estrogen concentration after pregnancy or after short-term oral contraceptive use [6]. Similarly, cervical tumors induced in K14-E7 mice stop and revert their growth in estrogen absence [6]. In addition, we informed previously in this animal model high BrdU incorporation in several cell layers of cervical squamous epithelium [24], while Ki-67 and cyclin E were overexpressed [23]. These are well-established proliferation markers which were positive in all cervical epithelium layers of high-grade squamous intraepithelial lesions and in invasive cancers developed in K14-E7 transgenic mice, indicating loss of cell cycle or epithelial growth control [23,24]. In this animal model, apoptosis was absent at any stage of cervical neoplastic progression in estrogen-treated K14-E7 transgenic mice [24]. Thus, in our model E7 plus estrogen could be blocking several tumor suppressor pathways, like the TGF-β pathway signaling to induce CC.

## Conclusion

Our observations indicate that loss of growth response to TGF-β in CC could originate from decreased cellular TGF-βRII expression because the lower TGF-βRII level in estrogen-treated K14-E7, as compared to that found in nontransgenic mice may contribute to cervical cancer. Additional studies are needed to find possible alterations of other components of this important signaling pathway and its biological relevance.

## Methods

### Transgenic mice

K14-E7 transgenic mice expressing the HPV16 E7 oncogene contain the HPV16 E6 and E7 translational open reading frames spanning nucleotides 79–883 positioned downstream of the human keratin 14 (K14) transcriptional promoter. K14-E6ttl/E7 (designated here as K14-E7) transgenic mice have a translation termination linker (ttl) in the E6 gene precluding E6 expression [47]. Animals were housed in a pathogen-free barrier facility. All experiments and procedures were carried out under an animal protocol approved by the University of Wisconsin IACUC.

### Hormone treatment

One-month old K14-E7 transgenic and nontransgenic virgin female mice were anesthetized with halothane, s.c. implanted in the dorsal skin with continuous release pellets delivering 0.05 mg 17β-estradiol over 60 days (Innovative Research of America, Sarasota, FL). Three estrogen pellets were administered in total during the 6 month period of estrogen treatment. A total of 40 mice were used. Ten mice each of Nt-E, Nt+E, E7-E and E7+E mice were employed in this study. Control mice received no pellet insertions.

### Tissue procurement and histology

One hour prior to sacrifice, mice were injected intraperitoneally with 100 μg/kg of bromodeoxyuridine (BrdU). At sacrifice, mice were anesthetized with 2.5% Avertin and perfused with 4% formaldehyde through the aorta. The reproductive tract, including the vagina, cervix, and uterine horns, was dissected and postfixed overnight at 4°C. The posterior vaginal wall was removed for orientation. Tissues were embedded in paraffin and were cut at ~100 μm intervals until the cervical canal was visible, after which 5 μm serial sections through the cervix were obtained. Every tenth section was stained with hematoxylin and eosin and evaluated for pathology. The treatment of these mice and the histopathological characterization of these tissues have been described previously [24].

### *In situ *RT-PCR

Detection of TGF-β2 and TGF-βRII mRNA was carried out using a two-step in situ RT-PCR procedure as previously reported [48] with the following modifications. Sections were pretreated with 0.5 μg/μl proteinase K (Sigma Aldrich; St Louis, MO) in 0.01 M PBS, pH 7.4, at room temperature for 30 min. After Proteinase K digestion, tissues were treated with 1 U/sample of DNase I RNase-free (Roche, U.S.A.) during 24 hrs at room temperature. After thoroughly washing with DEPC-treated water, reverse transcription was performed using the SuperScript II reverse transcriptase (Invitrogen, U.S.A.). TGF-β2 and TGF-βRII mRNA sequences obtained from [49] were used to design forward (5'-CCGCA-TCTCCTGCTAATGTTG-3') and reverse (5'-AATAGGCGGCATCCAAAGC-3') primers for TGF-β2 mRNA amplification. For TGF-βRII mRNA, primers 5'-AGCATCACGGCCATCTGTG-3' (forward) and 5'-TGGCAAACCGTCTCCAG-AGT-3' (reverse) were used (Invitrogen).

*In situ *RT reactions were performed by the application of 2.5 U Superscript™ II reverse transcriptase (Invitrogen, USA) to the slides. Positive controls consisted of in situ RT-PCR reactions in the absence of DNase and as a negative control buffer without Superscript™ II reverse transcriptase was applied to one section on each slide. Slides were incubated at 37°C for 2 h, then 94°C for 5 min. PCR amplification was performed using the corresponding primers and the system provided by Perkin Elmer. The slides were preheated to 70°C on the assembly tool included in the *in situ *Perkin Elmer equipment, 50 μl PCR master mix was added to each sample and the reaction was sealed using Amplicover discs and clips (Perkin Elmer, USA). After assembly, slides were placed at 70°C in the GeneAmp In situ PCR system 1000 (Perkin Elmer, USA) until running was started. Slides were incubated first at 94°C for 5 min (initial denaturation), followed by 20 cycles at 94°C for 1 min (denaturation), 60°C for 1 min (annealing) and 72°C for 1 min (extension), respectively. After PCR amplification, slides were washed for 5 min in 1× PBS pH 7.4, followed by 5 min in 100% ethanol before they were air dried. Slides were soaked in PBS containing 5% bovine serum albumin (Sigma, USA) for 30 min to block nonspecific binding activity (stringent wash). Immunohistochemical signal detection was carried out using mouse anti-digoxigenin monoclonal antibody Fab fragments conjugated to alkaline phosphatase (1:200 dilution, 30 min, room temperature) (Roche; Mannheim, Germany), and signals visualized by nitroblue tetrazolium chloride (NBT) and bromochloroindoxyl phosphate (BCIP) (Zymed, USA). We evaluated TGF-β2 and TGF-βRII mRNA in a total of 5 mice from each group.

### Real-time RT-PCR

Isolated RNA was controlled for quality by 2% agarose gel separation and ethidium bromide staining. RNA was quantified by spectrophotometry. Complementary DNA (cDNA) was synthesized using 2 μg of total RNA. The 20 μl reverse transcription reaction consisted of 2 μl 10× RT buffer, 0.5 mM each dNTP, 1 μM Oligo-dT primers, and 4 U Omniscript reverse transcriptase (QIAGEN, USA). The reverse transcription reaction was incubated for 1 h at 37°C and then at 93°C for 15 min. A no-template control was performed for each experiment, establishing the absence of genomic contamination in the samples. For the quantitative SYBR Green real-time PCR, 1 μl of each RT product was used per reaction and SYBR Green reaction was conducted using a QuantiTect™ SYBR Green PCR Reagents kit (QIAGEN, USA) and the protocol provided by the manufacturer. Optimization was performed for each gene-specific pair of primers prior to the experiment to confirm that 50 nM primer concentrations did not produce nonspecific primer-dimmer amplification signal in no-template control tube. Changes in fluorescence were recorded as the temperature was increased from 65°C to 95°C at a rate of 0.2°C/s to obtain a DNA melting curve. The characteristic peak at the melting temperature of the target product distinguishes it from amplification artefacts that melt at lower temperatures in broader peaks.

The primer sequences, that were designed using Primer Express Software, confirmed specificity of the PCR. TGF-β2, TGF-βRII, c-myc, p15 and gapdh mRNA sequences obtained from [49] were used to design forward and reverse primers. For TGF-β2 mRNA amplification we used (5'-CCGCATCTCCTGCTAATGTTG-3') (forward) and (5'-AATAGGCGGCATCCAAAGC-3') (reverse). For TGF-βRII mRNA, primers 5'-AGCATCACGGCCATCTGTG-3' (forward) and 5'-TGGCAAACCGTCTCCAGAGT-3' (reverse). For c-myc mRNA, primers 5'-TGCATTGACCCCTCAGTGGT-3' (forward) and 5'-TCCGAGGAAGGAGAG-AAGGC-3' (reverse). For p15 mRNA, primers 5'-TCTGCAGCTGGATCTGGTCC-3' (forward) and 5'-TCCTGAAAGGTAGAGGGCCC-3' (reverse). For gapdh mRNA, primers 5'-CATCTCCTCCCGTTCTGCC-3' (forward) and 5'-GTGGTG-CAGGATGCATTGC-3' (reverse). Each sample was tested in triplicate with quantitative PCR, and for standardisation of gene expression levels, mRNA ratios relative to the house-keeping gene gapdh were calculated. We evaluated TGF-β2 and TGF-βRII mRNA in a total of 3 mice from each group.

### Data analysis using 2^-ΔΔCT ^method

Real-time PCR was performed on the corresponding cDNA synthesized from each sample. The data were analysed using the equation described by Livak [50] as follows: Amount of target = 2^-ΔΔCT^. The threshold cycle (CT) indicates the fractional number at which the amount of amplified target reaches a fixed threshold. ΔCT = (average TGF-β2, TGF-βRII, c-myc and p15 CT – average gapdh CT). ΔΔCT = (average ΔCT untreated Nt mice (calibrator) – average ΔCT untreated or estrogen-treated mice). Validation of the method was performed as previously reported [51].

### Statistical analysis

Data are presented as mean ± standard deviation (S.D.). Statistical evaluation of significant differences was performed using the Student's *t*-test. Differences of P < 0.05 were considered statistically significant.

### Immunohistochemistry

Sections of 5 μm width were placed on poly-L-lysine-coated slides. Following deparaffination, the sections were immersed in an antigen-retrieval solution (DAKO, Glostrup, Denmark) for 40 min at 98°C. Endogen peroxidase was blocked with 3% H_2_O_2 _in absolute methanol, followed by immersion in a universal blocking reagent (Powerblock, Biogenex, San Ramon, CA, USA) for 10 min. The sections were incubated overnight at room temperature with rabbit polyclonal antibodies against TGF-β2 or TGF-βRII (Santa Cruz Laboratory, Santa Cruz, CA, USA) diluted 1/50 in PBS. Bound antibodies were detected with goat antirabbit immunoglobulin G labelled with peroxidase diluted 1/150 in PBS and the site of antibody binding was visualized using diaminobenzidine reagent. The slides were counterstained with Mayer's Hematoxylin (Sigma Diagnostics). We evaluated TGF-β2 and TGF-βRII protein levels in a total of 5 mice from each group.

## Abbreviations

HR-HPV, High risk human papilloma virus; TGF-β2, transforming growth factor beta 2; TGF-βRII, transforming growth factor beta type II receptor; CIN, cervical intraepithelial neoplasia; LSIL, low-grade squamous intraepithelial lesion; CIS, carcinoma in situ; CC, cervical cancer; BrdU, bromodeoxyuridine; ttl, translation termination linker; K14, keratin-14.

## Competing interests

The author(s) declare that they have no competing interests.

## Authors' contributions

**DCJ**: carried out all the molecular studies in nontransgenic and K14-E7 mice, and drafted the manuscript and participated in the design of the study; **HPR**: carried out histopathological analysis in the cervical samples from nontransgenic and K14-E7 mice and was involved in the revising of the manuscript critically; **LPF**: provided us nontransgenic and K14-E7 mice and samples, and revised critically the manuscript for important intellectual content; **GP**: conceived the collaborative study, participated in its design and coordination, helped to draft the manuscript and was involved in the revising of the manuscript critically for important intellectual content. All authors read and approved the final manuscript.
